# The Brazilian experience of implementing the active pharmacovigilance of dolutegravir

**DOI:** 10.1097/MD.0000000000014828

**Published:** 2019-03-08

**Authors:** Cynthia Julia Braga Batista, Renato Girade Correa, Livia Ramalho Evangelista, Karen Fleck, Leandro Silva, Francoise Renaud, Marco Vitoria, Meg Doherty, Adele Schwartz Benzaken

**Affiliations:** a Department of Surveillance, Prevention and Control of Sexually Transmitted Infections, HIV/AIDS and Viral Hepatitis, Ministry of Health, Brasilia; b Pharmacovigilance Office, GFARM - Brazilian Health Regulatory Agency, Anvisa, Brazil; c HIV Department, World Health Organization, Geneva, Switzerland.

**Keywords:** adverse reactions to drugs, AIDS, dolutegravir, HIV, pharmacovigilance

## Abstract

In 2017, the Ministry of Health Brazilian started using dolutegravir (DTG) 50 mg to all people living with HIV who began antiretroviral therapy (ART) or rescue regimens. Although DTG is thought to have better tolerability levels and a lower possibility of causing adverse reactions, it is necessary to continuously evaluate the safety profile of the drug in the population. Therefore, an active pharmacovigilance project for DTG was implemented. The objective of this study was to describe the Brazilian experience of implementing pharmacovigilance and the results obtained during the period between April and December 2017.

Active pharmacovigilance was implemented through patient interviews and an online questionnaire developed in the Medication Logistics Control System (SICLOM).

Of the total number of people on DTG in Brazil (79,742) 90.33% participated in the project, and 2.24% of those who participated reported adverse reactions to the drug; of those who reported adverse reactions, 73.86% were on first-line ART regimens, and 26.13% were on third-line regimens. The mean age of the patients who had adverse reactions to DTG was 39 years; 68.79% were male, and 31.21% were female. Of the adverse reactions reported, 50.39% were considered persistent. The 3 most frequent reactions were nausea (13.34%), diarrhea (9.83%), and headaches (9.23%).

The Brazilian experience with this project has been deemed successful by federal and local managers, and the online tool to collect data has proved to be an important strategy for the pharmacovigilance of DTG as well as that of other drugs.

## 1 Introduction

In 2017, in agreement with the Brazilian policy of universal and free-of-charge access to of the Unified Health Services (SUS), the Department of STIs, AIDS and Viral Hepatitis (DIAVH) of the Ministry of Health started using dolutegravir (DTG) to people living with HIV who initiate antiretroviral therapy (ART), except to pregnant women and those co-infected with tuberculosis, and in those using salvage regimens, as a substitution for raltegravir.

DTG, an integrase inhibitor, is indicated in combination with other ARVs for the treatment of HIV in adults and children older than 12 years because it presents better tolerance levels, is a regimen with long-term durability, has a high genetic barrier, involves a single daily dose, results in fast viral suppression, has fewer drug interactions, and has a lower probability of adverse reactions.^[1–4]^

Although clinical studies are important and necessary, no trial is exhaustive. There will always be the need for continuous safety profile evaluations of a drug in the community during its use in clinical practice. It is in clinical practice that exposure to other medications occurs, that the effects of this exposure on pre-existing conditions may be observed, and that the medication is used for longer periods of time. It is in these situations, therefore, that rarer – and sometimes severe – adverse reactions may appear. In this context, pharmacovigilance plays an important role.

According to the World Health Organization (WHO), pharmacovigilance is defined as “the science and activities related to the identification, evaluation, understanding, and prevention of adverse reactions or any other problems relative to the use of medications”.^[5]^

Active pharmacovigilance methods play an extremely important role that is complementary to that of spontaneous notifications because pharmacovigilance provides pertinent data about unique populations and specific drugs. Data about drug consumption and medication use are essential to assess the safety of the product. It is imperative to promote this type of programmed study to improve patient safety. Additionally, it is recommended that these studies be implemented in conjunction with a spontaneous notification system.^[6]^

In some countries, adverse reactions to drugs are between the fourth and sixth leading causes of death, highlighting the need for pharmacovigilance programs.^[6]^

Side effects and central nervous system disorders related to the use of integrase inhibitors in ART have been reported since the introduction of such drugs. The most common reactions identified at the start of DTG therapy were nausea, headaches, diarrhea, and sleep disorders.^[7]^

In Brazil, ART is provided free of charge to all people diagnosed with HIV/AIDS. This is protected by Federal Law 9313, dated November 13, 1996. Since then, the government has taken charge of the procurement and distribution of these drugs, and they are not available in private pharmacies.

Considering the continental dimensions of Brazil, the regional differences, the high number of Drug Dispensation Units (UDM), and the large number of patients who start ART annually as well as the need to organize medication logistics and to clinically follow up with patients, the Medication Logistics Control System (SICLOM) was implemented in 1997. This computer-based system with online access is used by 866 UDMs all over the country who record all drug dispensations. It also contains patient-centered clinical information such as identification, gender, age, skin color, race, the doctor in charge of follow-up, dispensation history, utilized regimens, the viral load, the CD4 count, and all the logistics controlling each medication.

The rules, established through the Clinical Protocols and Therapeutic Guidelines (CPTG) for the management of HIV infections in adults, children, and pregnant women, are also included in SICLOM, preventing the prescription of regimens that have not been standardized by CPTG. This procedure also reduces dispensation mistakes, increases ART safety, allows for a more efficient control of logistics, and permits the monitoring of the treatment or the promoting of transitions to new therapies.

The use of SICLOM database has facilitated monitoring and directed public policies towards achieving the 90-90-90 goals that were established by the Joint United Nations Programme on HIV/AIDS (UNAIDS)^[8]^ and adopted in Brazil since 2014.

The active pharmacovigilance project was implemented in the SICLOM and was designed with the objectives of improving knowledge of the safety of DTG. Identifying the occurrence and nature of previously unknown side effects as well as any deviations from the expected frequency of known adverse reactions to this medication and determining the impact of adverse reactions on adherence to ART, regimen substitution and treatment outcomes.

The purpose of this study was

1. to describe the planning and implementation process of the active pharmacovigilance of DTG project in 2017, when the new ARV was introduced to the Brazilian public health network, free of charge, and integrated into the national pharmacovigilance system,
2. to present the initial results obtained between April and December 2017.

## 2 Methods

### 2.1 Project planning

The Brazilian National Health Surveillance Agency (ANVISA) has an online system to receive notifications of incidents, adverse reactions, and technical complaints related to the use of products and services controlled by sanitary surveillance. This spontaneous notification system allows institution/company professionals to report adverse events to medications by signing in and filling out the specific form.

Because SICLOM is used for 100% of the ARV dispensations at UDMs, this system is provided with individualized information for each patient whenever the medication is dispensed. Therefore, we opted to implement the active pharmacovigilance of DTG project using interviews and online questionnaires filled out at the second dispensation and reported through the SICLOM system. This facilitated data collection, since UDM professionals all over the country already use the system and would not need to access another site to record the information related to the notifiers and the patients.

The survey questionnaire was designed by a group of professionals from DIAVH and ANVISA with the collaboration of experts from the WHO. The pharmacist who dispensed the medication and/or the doctor who prescribed it were considered the main interviewers. Printing the questionnaire at the health center to allow the patient to fill it out at home was also allowed. In that case, the patient would return the completed questionnaire when he or she returned to the health center for a new dispensation.

The questionnaire includes questions about the type and duration of the adverse reaction to medication (the WHO standard list with 5000 options), the severity of the reaction, clinical data prior to the use of medication, manifestations of immune reconstitution inflammatory syndrome, previous or active opportunistic diseases aggravated after initiating DTG treatment, and the use of medications other than the ARV (including herbal medicines and multivitamins), as shown in Table 1 . The inclusion of specific adverse reactions was based on international studies.

**Table 1:**
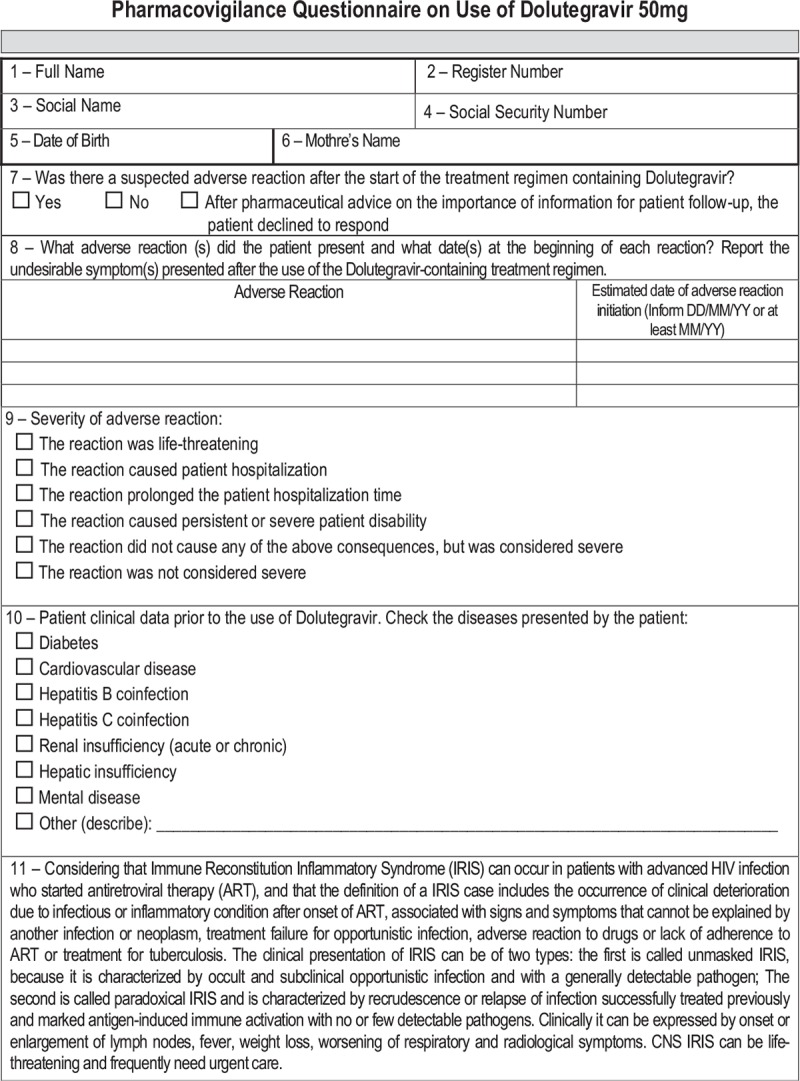
Patient survey questionnaire – active pharmacovigilance of DTG project, Brazil, 2017.

**Table 1 (Continued):**
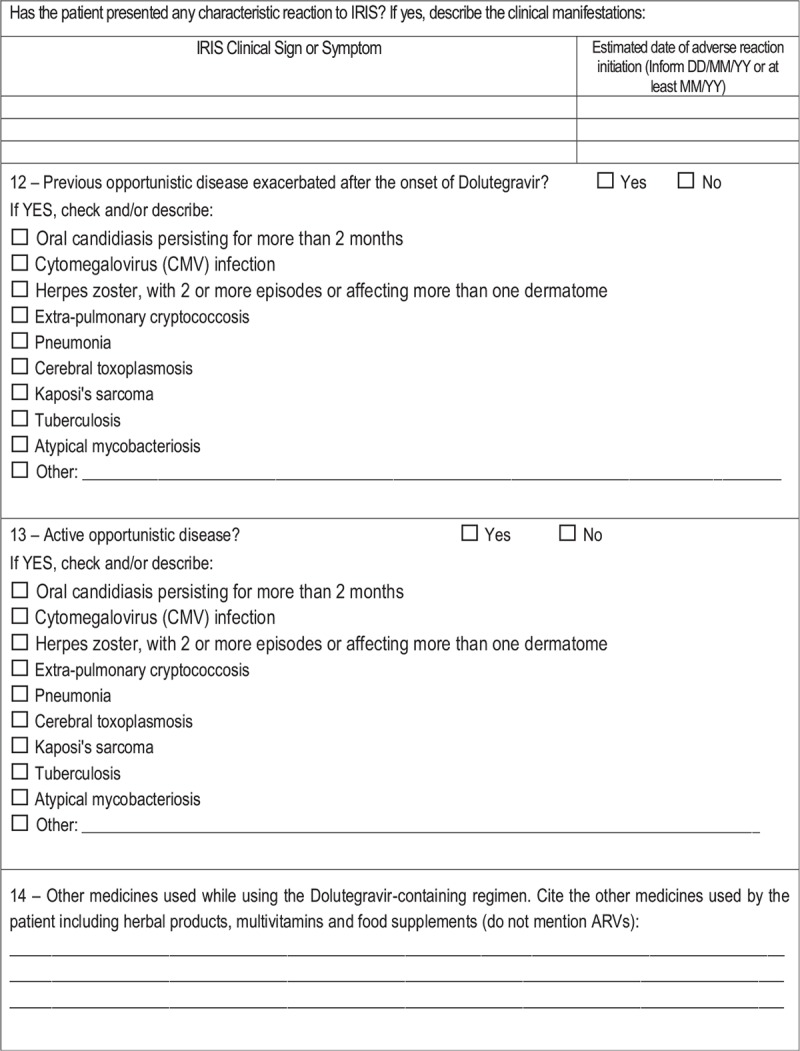
Patient survey questionnaire – active pharmacovigilance of DTG project, Brazil, 2017.

Most of the pharmacies that dispense ARVs have a limited number of employees and a large number of patients. Therefore, the following strategies were planned to facilitate filling out the questionnaire:

1. *Self-Complete* system for questions 8 and 11 (for which there are numerous options), according to the standard list of the WHO. This system allows the name of the reactions to be filled in by typing just 1 or 2 letters of the side effect reported by the patient.
2. Provision of closed fields for questions 9, 10, 12, and 13 by listing main occurrences.

### 2.2 Questionnaire validation

To design a user-friendly questionnaire that would meet the needs of all the professionals involved in the project, a pilot project was implemented in 10 ARV UDMs.

The selection criteria for the health centers involved in the pilot project were as follows:

1. the number of new patients who started treatment the previous year;
2. the regional location; and
3. the availability of professionals.

After the selection, the health centers were organized into 3 random groups according to the amount of available time professionals had at each site. Videoconferences were then conducted to discuss the implementation of the project and to share the questionnaire model.

The following professionals were involved in all states: STI/AIDS local managers/coordinators, pharmacists, supervisors, and dispensers. Meetings were held during the second fortnight of March 2017, and the main topics addressed were the following:

1. The feasibility of the implementation of the project in organizations with large numbers of patients;
2. The perception by pharmacists of the importance of the project;
3. A joint evaluation of the questionnaire proposed to capture the data;
4. The definition of the pilot project operating period;
5. The initial contact of patients by the professional in charge of filling out the questionnaire.

The professionals and managers involved considered the project viable and relevant. It was favorably received, especially by the pharmacists who dispense ARVs. The pilot project was initiated on April 2017 and lasted until June 2017.

To monitor the use of the questionnaire, we asked professionals to provide feedback via email on the use of the system tool after the first month of implementation of the pilot project. The main suggestions for improvements were the following:

1. To implement an alert for the finalization of the questionnaire to avoid there being any doubts that all information was saved;
2. To include a field to classify reactions as persistent (i.e., associated with substantial disruption of ability of a person to conduct normal life functions) or not;
3. To include 1 more option in the questionnaire: “after instructions, the patient will return the filled-out questionnaire at the next dispensation”;
4. To develop a management report for the health center to report on the data from the questionnaires filled out at the center;
5. To implement the possibility of altering the data after finalizing the questionnaire.

Suggestions 3 and 5 were not accepted by the tool managers due to data quality concerns and the risk of data loss, as well as the possibility of daily alterations of data (suggestion 5) and the event of a patient not returning the filled-out questionnaire at the next dispensation (suggestion 3). Suggestions 1, 2, and 4 were approved and implemented.

### 2.3 Project scale up

The project was rolled out to 866 pharmacies dispensing ARVs after the implementation of the suggestions for improvement and its wide dissemination among the participants.

After the expansion, monthly and systematic data analysis resulted in improvements of the questionnaire to qualify information and allow data tabulation. The main alterations were the following:

1. The removal of the field “others” from question 10 referring to previous clinical data relative to DTG because it was observed that the selection of the field occurred randomly without specifying or qualifying the information;
2. Making it mandatory to fill out the start and end dates of reactions in questions 8 and 11, with automatic data checking to verify if the end date of reaction was included when option “Yes” was selected in the field related to the existence of a persistent reaction;
3. The inclusion of a question requesting the following information about the person who filled out the questionnaire:
  a. pharmacist or another UDM professional, at the moment of dispensation or after interview with patient;
  b. patient, at the moment of ARV dispensation, or the patient returned the questionnaire, filled out by him/herself or a family member;
  c. doctor, during medical examination;
  d. nurse at the health center to which the patient is linked.

The data analyzed in this study were collected during the period between April and December 2017.

## 3 Results

Out of the 79,742 people receiving DTG in Brazil at the time of this study, 50,487 (63.31%) were on first-line regimens and 29,255 (36.68%) were on third-line ART; 72,032 (90.33%) of the people receiving DTG participated in the pharmacovigilance project, and 1,072 (1.34%) refused to participate. Of the total number of patients who participated in the project, 1615 (2.24%) had adverse reactions. Of those who experienced adverse reactions, 1193 (73.86%) were on first-line ART, and 422 (26.13%) were on third-line regimens. During the study, there were no deaths reported related to an adverse reaction to the use of DTG.

**Figure 1:**
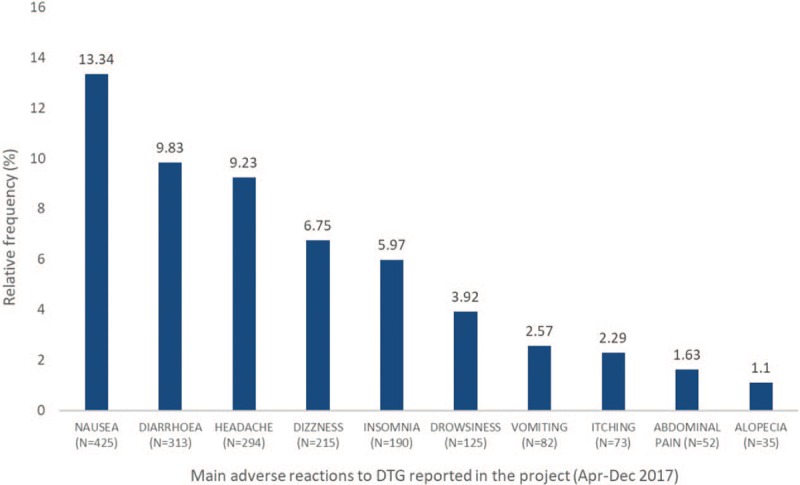
Main adverse reactions reported via SICLOM during the DTG pharmacovigilance project and their relative frequency (%).SICLOM = Medication Logistics Control System, DTG = dolutegravir.

During the analyzed period, 3185 adverse reactions to medication were reported. The relative frequency of the 10 most common reactions are shown in Figure 1.

Of the total reported reactions, 1605 (50.39%) were considered persistent.

Of the 1615 patients who reported adverse reactions, 1433 (88.73%) reported that they were not serious, 11 (0.68%) said the reaction put their lives at risk, 16 (0.99%) reported that the reaction caused persistent or severe disability, 23 (1.42%) declared that the reaction led to hospitalization, and 138 (8.54%) reported that the reaction did not lead to hospitalization, disability, or put their lives at risk but was nevertheless considered serious. There were no reports that the reaction lasted longer than the patient's hospitalization time.

Existing opportunistic diseases prior to the use of DTG were present in 34 (2.10%) of the patients who reported adverse reactions, and after start to the use DTG 55 (3.40%) patients reported active opportunistic diseases. Specific studies are being conducted with these patients.

Up to December 2017, 149 people had substituted DTG in the ART due to adverse reactions.

## 4 Discussion

The strategies used by the STI/AIDS Department to implement the active pharmacovigilance of DTG project were considered effective, providing satisfactory results and contributing to rolling out the project to include other ARVs. Other benefits derived from this experience are highlighted below:

1. The integration between the STI/AIDS Department and ANVISA for a joint design of the tool;
2. The use of SICLOM, a system that is well-established and well-accepted by the professionals who dispense ARVs all over Brazil;
3. The interest of pharmacists who reported (during videoconferences) an increased sense of self-worth;
4. The importance of involving managers, supervisors and local coordinators in the process to support the professionals in charge of the interviews;
5. The implementation of the pilot project, allowing professionals who dispense ARV to participate in the evaluation and to contribute to adjusting the tool to fit the reality experienced by their organizations;
6. The establishment of a deadline to receive suggestions/feedback after the first month of using the tool;
7. The development of reports to allow local information monitoring at each health center;
8. Periodic data analysis was essential to the implementation of improvements in information quality and data tabulation.

The results obtained during the 8-month project (Apr/Dec 2017) demonstrated that the profile of adverse reactions identified by the active pharmacovigilance project in Brazil are similar to the reactions described as very common (more than 10% of patients) or common (between 1% and 10% of patients) in the package insert.^[9]^

A comparison was also made between the information obtained by this project with the data recorded in the world database of the Medication Monitoring Centre (Uppsala Monitoring Centre), headquartered in Uppsala. Up to the moment of the analysis, there had been 1754 spontaneous notifications of adverse reactions to DTG.^[10]^

There are some differences between the reactions recorded in Uppsala and the most common reactions reported in Brazil. This may be related to the fact that the therapeutic regimens used in other countries may not be the same as the 1 adopted in our country and that the profile of patients on DTG may be different. Additionally, these differences may result from our use of spontaneous notifications. However, the profile of reactions recorded by the Uppsala Monitoring Centre^[10]^ is similar to that found in the literature and in the Brazilian monitoring process.

In relation to the number of people who substituted DTG in ART due to adverse reactions, it is important to note that, based on the data collected, nausea, diarrhea, and headaches (adverse reactions presented as reasons for changing the therapeutic regimen) are described as very common side effects in the drug package insert; dizziness, insomnia, vomiting, and abdominal pain are commonly associated with using DTG (1–10%); and hypersensitivity reactions occur at lower frequencies and are considered uncommon (0.1%–1%).^[9]^ Attention should also be drawn to the fact that these more frequent adverse reactions may be classified as not being severe. The profiles of the adverse reactions observed in this study are in accordance with the safety profile presented in the DTG clinical trials. This confirms what is known about the drug and, consequently, leads to greater patient safety.

It should also be mentioned that, according to the WHO, an adverse reaction to medication is defined as a harmful and non-intentional response to the use of medication, occurring with doses normally used in human beings for the prophylaxis, diagnosis, or treatment of diseases. Thus, it should be kept in mind that all medications present risks associated with their usage. Since adverse reactions are part of the risks associated with the consumption of a drug, it is not normally possible to anticipate situations where the use of a drug is advisable or not, based on only adverse reactions. In principle, an adverse reaction does not necessarily mean that there is a problem with a specific drug. The fact that side effects not considered serious (such as headaches, nausea, and vomiting) may lead to the interruption of a treatment points to the need for their assessment by healthcare professionals and by the SUS, because these reactions influence – in the case of this study – the therapeutic regimen that will be prescribed for a specific patient and the treatment outcomes. In certain situations, as shown in this research, people substitute the use of the drug with another because of non-serious reactions. Further studies are needed to document potential risk factors associated with drug reactions in people receiving DTG as part of the ART regimen.

In conclusion:

- The Brazilian experience of implementing the active pharmacovigilance of DTG was considered successful by federal (DIAHV and ANVISA) and local managers.
- Brazil is structurally prepared to monitor and manage toxicities related to any ARV, considering the use of SICLOM by all ARV dispensing units. However, this would not be possible immediately for other drugs due to the limitation of systems used for dispensing, which are not computerized in their entirety.
- A total of 72,032 patients participated in the project in the period between April and December 2017.
- Of those patients who participated, 2.24% reported some type of adverse reaction to DTG.
- Of those patients who reported an adverse reaction, 73.86% were on first-line ART and 26.13% were on third-line regimens.
- A total of 3185 adverse reactions were reported during the study period, of which 13.34% were related to nausea, 9.83% were related to diarrhea, and 9.23% were related to headache.
- Of the adverse reactions reported, 50.39% were considered persistent.
- In total, 149 patients substituted DTG in their ART due to adverse reactions.
- The reactions reported in Brazil are like the reactions recorded in clinical trials and in international pharmacovigilance data.

## Acknowledgments

Alexsana Sposito Tresse; Robério Alves Carneiro Júnior; Juliana Monteiro Cruz; Karim Sakita, Gláucio Mosimann Júnior and Eduardo Malheiros for their contributions.

## Author contributions

**Writing – review & editing:** Cynthia Julia Braga Batista, Renato Girade Correa, Livia Ramalho Evangelista, Karen Fleck, Leandro Silva, Francoise Renaud, Marco Vitoria, Meg Doherty, Adele Schwartz Benzaken.

## References

[1] Walmsley S Baumgarten A Berenguer J Dolutegravir plus abacavir/lamivudine for the treatment of HIV-1 infection in antiretroviral therapy-naive patients: week 96 and week 144 results from the SINGLE randomized clinical trial. J Acquir Immune Defic Syndr 2015;70:515–9. doi:10.1097/QAI.0000000000000790. 26262777 10.1097/QAI.0000000000000790 PMC4645960

[2] Molina J-M Clotet B van Lunzen J Once-daily dolutegravir is superior to once-daily darunavir/ritonavir in treatment-naïve HIV-1-positive individuals: 96 week results from FLAMINGO. J Int AIDS Soc 2014;17 4Suppl 3:114915 doi:10.7448/IAS.17.4.19490. 10.7448/IAS.17.4.19490 PMC4224885 25393999

[3] Raffi F Jaeger H Quiros-Roldan E Once-daily dolutegravir versus twice-daily raltegravir in antiretroviral-naive adults with HIV-1 infection (SPRING-2 study): 96 week results from a randomised, double-blind, non-inferiority trial. Lancet Infect Dis 2013;13:927–35. doi:10.1016/S1473-3099(13)70257-3. 24074642 10.1016/S1473-3099(13)70257-3

[4] Cahn P Pozniak AL Mingrone H Dolutegravir versus raltegravir in antiretroviral-experienced, integrase-inhibitor-naive adults with HIV: week 48 results from the randomised, double-blind, non-inferiority SAILING study. Lancet 2013;382:700–8. doi:10.1016/S0140-6736(13)61221-0. 23830355 10.1016/S0140-6736(13)61221-0

[5] WHO. Transition to new antiretrovirals in HIV programmes. WHO. 2017 http://www.who.int/hiv/pub/toolkits/transition-to-new-arv/en/ Accessed February 25, 2019.

[6] Pan American Health Organization. Good Pharmacovigilance Practices for the Americas; 2011 http://apps.who.int/medicinedocs/documents/s18625en/s18625en.pdf Accessed February 25, 2019.

[7] Taha H Das A Das S Clinical effectiveness of dolutegravir in the treatment of HIV/AIDS. Infect Drug Resist 2015;8:339–52. doi:10.2147/IDR.S68396. 26491363 10.2147/IDR.S68396 PMC4598225

[8] Global Aids Response Progress Reporting 2015: Geneva: WHO and UNAIDS; 2015 http://www.unaids.org/sites/default/files/media_asset/JC2702_GARPR2015guidelines_en.pdf Accessed February 25, 2019.

[9] Tivicay 50 mg - GlaxoSmithKline Brasil Ltda. http://www.anvisa.gov.br/datavisa/fila_bula/frmVisualizarBula.asp?pNuTransacao=24633662016&pIdAnexo=4000318 Accessed February 25, 2019.

[10] WHO Programme for International Drug Monitoring. Uppsala Monitoring Centre. https://www.who-umc.org/ [accessed August 20, 2003].

